# Tracheostomy in children: a narrative review

**DOI:** 10.3389/fsurg.2026.1787750

**Published:** 2026-04-01

**Authors:** Maria Vargas, Giuseppe Servillo

**Affiliations:** Department of Neurologic, Reproductive and Odontostomatological Sciences, University of Naples Federico II, Naples, Italy

**Keywords:** children, critical care, epidemiology, review, tracheostomy

## Abstract

Tracheostomy, a surgical procedure creating an artificial airway in the trachea, has evolved significantly since 1999 in pediatric care, driven by technological advancements and a deeper understanding of pediatric respiratory physiology. While once reserved for emergency situations or as a last resort for airway obstruction, its role has expanded to encompass long-term respiratory support, chronic aspiration management, and improved quality of life for children with complex medical conditions. The aim of this review, although not systematic, is to provide insight for pediatric surgeons, pulmonologists, critical care physicians, and other healthcare professionals involved in the care of children with tracheostomies.

## Introduction

Tracheostomy, a surgical procedure creating an artificial airway in the trachea, has evolved significantly since 1999 in pediatric care, driven by technological advancements and a deeper understanding of pediatric respiratory physiology (1). While once reserved for emergency situations or as a last resort for airway obstruction, its role has expanded to encompass long-term respiratory support, chronic aspiration management, and improved quality of life for children with complex medical conditions (1). The decision-making process surrounding tracheostomy in children is multifaceted, influenced by the underlying etiology, the child's overall health status, and the availability of specialized care (2). Despite its increased utilization, a standardized approach to tracheostomy management in pediatrics remains elusive, with practices varying across institutions and individual practitioners (3).

### Background of tracheostomy in children

Tracheostomy in the pediatric population presents unique challenges compared to adults, owing to anatomical and physiological differences. The pediatric airway is smaller and more pliable, making it susceptible to complications such as tracheal stenosis and granulation tissue formation. Furthermore, children have a higher metabolic rate and oxygen consumption, rendering them more vulnerable to respiratory compromise (4). The selection of appropriate tracheostomy tube size and type is crucial in children to minimize airway trauma and ensure adequate ventilation. Cuffless tubes are generally preferred in children to reduce the risk of tracheal injury, while cuffed tubes may be necessary in specific situations, such as during positive pressure ventilation or to prevent aspiration (5). The psychological impact of tracheostomy on children and their families should not be underestimated. Comprehensive support services, including education, counseling, and peer support groups, are essential to promote adaptation and improve quality of life (5).

The evolving landscape of pediatric critical care has witnessed a significant shift in the utilization of tracheostomy. The advent of non-invasive ventilation has altered the need for tracheostomies in children (6). Improved survival rates among premature infants and children with chronic illnesses have led to an increased population of tracheostomy-dependent children requiring long-term care. The increase in tracheostomy use in mechanically ventilated patients shows a peak in 2008 but has declined since then (7). Advances in surgical techniques, such as minimally invasive approaches and improved perioperative care, have contributed to a reduction in tracheostomy-related complications. However, even when performed by expert surgeons, tracheostomies are associated with complications that need close attention to surgical technique (8).

### Purpose and scope of the review

This narrative review aims to provide a comprehensive overview of tracheostomy in children, encompassing its indications, techniques, complications, and long-term management. The review will also address the evolving role of tracheostomy in the context of contemporary pediatric critical care and explore future directions for research and innovation in this field. The aim is to provide insight for pediatric surgeons, pulmonologists, critical care physicians, and other healthcare professionals involved in the care of children with tracheostomies, even if this review did not use a systematic approach but a narrative approach from the point of view of the experts in the field of tracheostomy.

## Methods

A comprehensive literature search was conducted using databases such as PubMed, Embase, and Cochrane Library. Search terms included “tracheostomy,” “pediatrics,” “children,” “indications,” “complications,” “management,” and related keywords. The search was limited to articles published in English. Articles referring to pediatric patients were included in this study (age ≤ 18 years old). Relevant articles were selected

(1) On the base of their relevance to the scope of the review, with a focus on studies that provided original data or synthesized existing evidence,
(2) With the purpose to overview of tracheostomy in children, encompassing its indications, techniques, complications, and long-term management.

Articles were excluded if they focused exclusively on adult populations or were not relevant to the topic of tracheostomy in children.

## Epidemiology of tracheostomy by age

The indications for tracheostomy in children vary depending on age. In neonates and infants, tracheostomy is most performed for congenital airway abnormalities, such as laryngeal or tracheal stenosis, or for prolonged mechanical ventilation due to prematurity or respiratory distress syndrome (9). In older children, tracheostomy may be necessary for acquired conditions, such as trauma, infection, or airway obstruction. The incidence of tracheostomy for prolonged mechanical ventilation increased across all age groups, most significantly among patients younger than 55 years (9). Studies have shown that certain age groups, such as infants and children with neuromuscular disorders, are at higher risk for tracheostomy-related complications. Moreover, pediatric patients are underrepresented in dysphagia research, even as dysphagia is prevalent in children with neurological impairment (10, 11). Neurologically impaired children often experience esophageal dysmotility, which is almost universally seen in children who have undergone surgery to repair esophageal atresia (12).

### Italian data from ministry of health

Individual patient data were requested to the section of the discharge report of the Italian Ministry of Health (National Archive for Hospital Discharge Form, Ministry of Health) about tracheostomy categorized by the codes 541 and 542 from the 2009 to 2014. Accordingly, we found that the code 541 (tracheostomy) was associated with 96 h of mechanical ventilation and major surgery, and the code 542 (tracheostomy) was associated with 96 h of mechanical ventilation without major surgery.

A total of 2,572 tracheostomy were performed in pediatric patients from 2009 to 2014. Tracheostomy were performed more often in bays than in girls. The age of tracheotomized children was decreasing during the years while he length of stay was increasing. Tracheostomy rate per 100,000 child-year was no different during the time. Table 1 showed the characteristics of the pediatric patients included in this evaluation.

**Table 1:** Characteristics of the pediatric patients.

Years	2009	2010	2011	2012	2013	2014	*p*
Tracheostomy (*n*)	408	438	476	434	408	408	0.54582
Sex							
Male	245	274	300	257	255	245	1
Female	163	164	176	177	153	163	1
Rate of rate per 100,000 child-years	3.78	4.04	4.39	4.10	3.85	3.80	
Age	9.308411 ± 7.42986	8.238318 ± 7.659723	7.004662 ± 7.400727	6.642523 ± 7.19084	7.580882 ± 7.383751286	6.313725 ± 7.236615	
Length of stay, median IQR	35 (20–63)*	39 (20.25–77.75)	39 (20–78)	46 (24–85)*	40 (22–79.25)	41 (23–77)	0.01578
Total cost for 1 day	2,077,481	2,398,424	2,030,363	531,549	605,656	823,063	0.25966

Data were received from the discharge report of the Italian Ministry of Health) and referred to a perind from the 2009 to 2014 years.

* *p* < 0.01.

**Figure 1:**
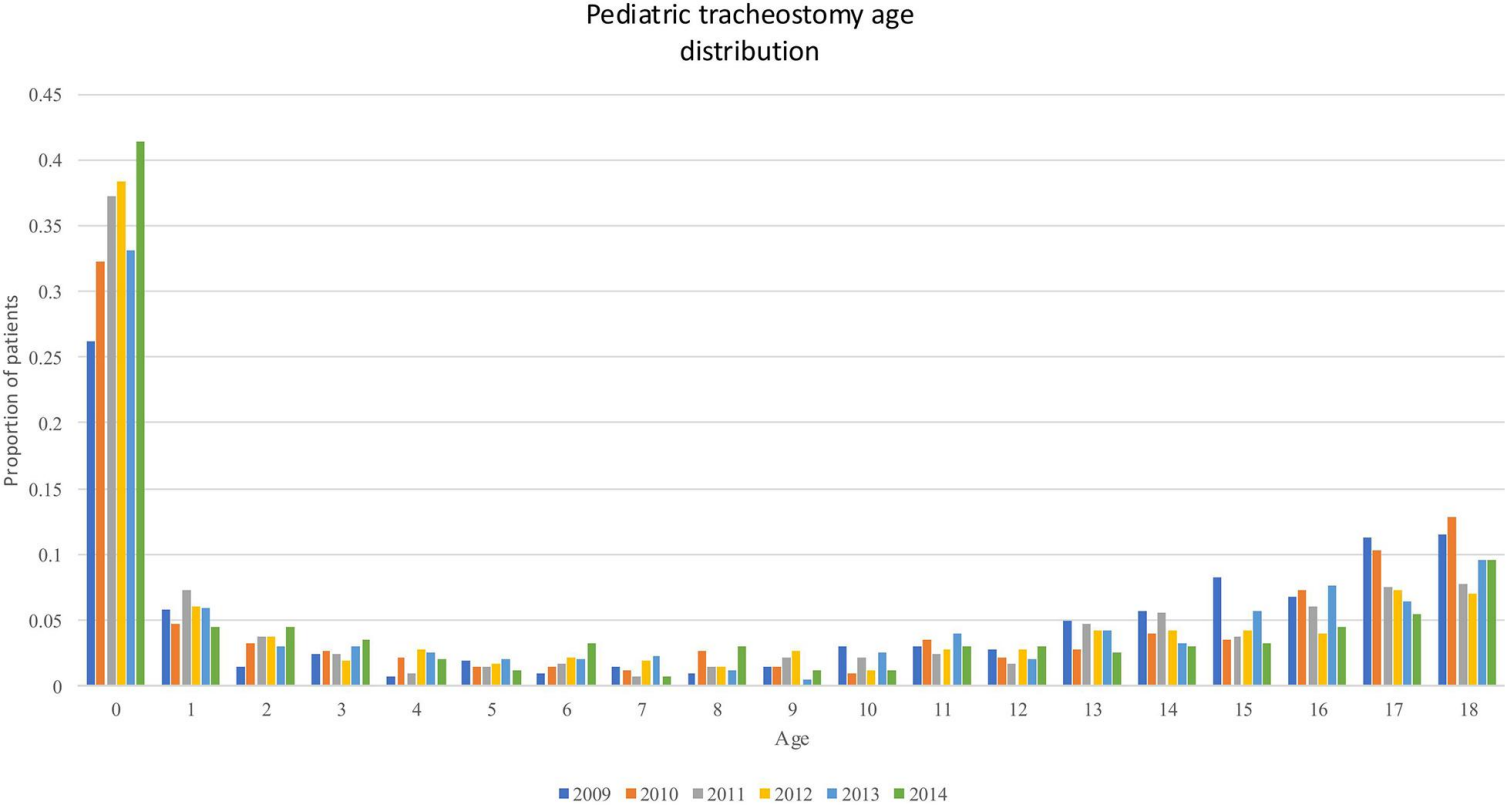
.

Interestingly, while dividing the number of tracheostomies by years we found that they were mostly performed during the first year of life or during the adolescence. Figure 1 showed the numbers of tracheostomies by years from the 2009 to 2014.

### Indications for tracheostomy in children

#### Upper airway obstruction

Upper airway obstruction is a common indication for tracheostomy in children, encompassing a wide range of conditions that compromise the patency of the upper respiratory tract. Congenital anomalies, such as choanal atresia, Pierre Robin sequence, and laryngeal web, can cause significant airway obstruction in newborns and infants, often necessitating tracheostomy to establish a secure airway. Acquired conditions, including infections like epiglottitis, croup, and bacterial tracheitis, can lead to acute upper airway obstruction requiring emergent tracheostomy. Subglottic stenosis, a narrowing of the airway below the vocal cords, can be congenital or acquired, with the latter often resulting from prolonged endotracheal intubation (13). In such cases, tracheostomy may be required to bypass the obstructed airway and facilitate ventilation. External compression of the airway, such as from cervical masses or vascular anomalies, can also cause upper airway obstruction and warrant tracheostomy.

#### Prolonged mechanical ventilation

Tracheostomy is frequently performed in children who require prolonged mechanical ventilation due to respiratory failure or other underlying medical conditions. Neuromuscular disorders, such as spinal muscular atrophy and muscular dystrophy, can impair respiratory muscle function, leading to chronic respiratory insufficiency and the need for long-term ventilator support. Similarly, children with chronic lung diseases, such as bronchopulmonary dysplasia and cystic fibrosis, may require prolonged mechanical ventilation due to recurrent respiratory infections and progressive lung damage. Central nervous system disorders, such as traumatic brain injury and spinal cord injury, can also result in respiratory failure and the need for tracheostomy. Tracheostomy facilitates airway access for suctioning and pulmonary hygiene, reducing the risk of pneumonia and other respiratory complications.

#### Neuromuscular disorders

The decision to perform tracheostomy in children with neuromuscular disorders requires careful consideration of the patient's overall prognosis, functional status, and family preferences. Non-invasive ventilation and cough-assist devices can reduce the necessity for tracheostomy in certain neuromuscular disorders (14). Tracheostomy may be considered in children with progressive neuromuscular disorders who are at high risk for respiratory failure, aspiration, and recurrent pneumonia, with the goal of improving comfort, facilitating care, and prolonging survival (15). However, the timing of tracheostomy should be individualized based on the child's specific needs and the family's values.

#### Complex congenital and acquired tracheo-oesophageal fistulae

Multidisciplinary approach to complex cases is becoming increasingly common across all specialties and is important in making decisions in these difficult cases (16). Repair of long gap oesophageal atresia with or without tracheo-oesophageal fistula remains complex and controversial. Tracheostomy may be required when primary anastomosis is not feasible.

#### Congenital anomalies

Various congenital anomalies, including laryngeal clefts, tracheal agenesis, and tracheoesophageal fistulas, may necessitate tracheostomy to secure the airway and facilitate ventilation. Esophageal atresia, a condition in which the esophagus does not form a continuous passage to the stomach, is often associated with tracheoesophageal fistula, an abnormal connection between the trachea and the esophagus (17). In such cases, tracheostomy may be required to manage airway secretions and prevent aspiration (18, 19).

### Surgical vs. percutaneous tracheostomy

#### Open surgical tracheostomy

The open surgical technique involves making a transverse or vertical incision in the anterior neck, dissecting through the subcutaneous tissue and strap muscles to expose the trachea. A window is then created in the anterior tracheal wall, typically by excising a small portion of cartilage, and the tracheostomy tube is inserted and secured with sutures or ties. The open surgical technique allows for precise placement of the tracheostomy tube and is often preferred in infants and young children due to their smaller anatomy.

#### Percutaneous tracheostomy

Percutaneous tracheostomy is a minimally invasive technique that involves creating a tracheostomy using a series of dilators passed through a small skin incision. The Seldinger technique, which involves inserting a guidewire into the trachea under bronchoscopic guidance, is commonly used to facilitate the placement of the tracheostomy tube. Percutaneous tracheostomy is typically performed in older children and adolescents with favorable anatomy and without significant comorbidities (20).

### Complications of pediatric tracheostomy

#### Immediate complications

Immediate complications of tracheostomy can occur during the procedure or just after it and may include suffocations, cardiac arrest, tension pneumothorax, pneumomediastinum, hemothorax, massive bleeding.

#### Early complications

Early complications of tracheostomy can occur within the first few days or weeks after the procedure and may include bleeding, pneumothorax, infection, and accidental dislodgement of the tracheostomy tube. Bleeding can result from injury to blood vessels during the surgical procedure or from erosion of the trachea by the tracheostomy tube (21). Pneumothorax, or air leakage into the pleural space, can occur if the pleura is inadvertently punctured during the tracheostomy procedure. Infection can develop at the tracheostomy site or in the lower respiratory tract, leading to tracheitis, pneumonia, or sepsis. Accidental dislodgement of the tracheostomy tube can result in airway obstruction and respiratory distress, requiring immediate intervention. Over-inflation of the cuff is vital to exert pressure on the bleeding vessel, and the tracheostomy tube should not be removed, as it can cause aspiration and drowning in blood (22).

#### Late complications

Late complications of tracheostomy can occur months or years after the procedure and may include tracheal stenosis, tracheomalacia, tracheoesophageal fistula, and granuloma formation (23). Tracheal stenosis, or narrowing of the trachea, can result from scarring or inflammation caused by the tracheostomy tube (24). Tracheomalacia, or softening of the tracheal cartilage, can occur due to prolonged pressure from the tracheostomy tube. Tracheoesophageal fistula, an abnormal connection between the trachea and the esophagus, can develop because of erosion of the tracheal wall by the tracheostomy tube. Granuloma formation, or the development of inflammatory tissue around the tracheostomy site, can cause airway obstruction and difficulty with tracheostomy tube changes. Tracheocutaneous fistula is also a common complication in decannulated children (25).

#### Prevention and management of complications

To minimize the risk of complications, meticulous surgical technique, proper tracheostomy tube selection, and diligent postoperative care are essential. Regular monitoring of the tracheostomy site, humidification of inspired air, and frequent suctioning of secretions can help prevent infection and airway obstruction. In cases of bleeding, prompt identification of the source and appropriate interventions, such as cauterization or surgical repair, are necessary. Pneumothorax may require chest tube insertion to evacuate air from the pleural space. Accidental dislodgement of the tracheostomy tube requires immediate replacement of the tube to restore airway patency. Management of late complications may involve endoscopic procedures, such as dilation or laser therapy, or surgical reconstruction of the trachea (26).

In the rare event of tracheobronchial injury, prompt surgical intervention and endoscopic monitoring are crucial for a positive outcome (27). Laryngotracheal trauma management necessitates a well-defined algorithm to ensure airway patency and minimize long-term complications (28). Vigilance is needed as victims of significant laryngotracheal trauma may have other injuries that conceal airway injury (29, 30). The presence of shock, respiratory distress, a full stomach, maxillofacial trauma, neck hematoma, laryngeal disruption, cervical spine instability, and head injury all combine to increase tracheal intubation difficulty in the trauma patient (31). Iatrogenic cervical tracheal stenosis, which can arise post-intubation or post-tracheostomy, presents a significant challenge, though research into its causes and prevention has somewhat diminished since the 1960s (32). It is crucial to recognize the anatomical relationships in the area to circumvent many complications, as documented by annually emerging case reports (33). Early diagnosis of complete tracheal rupture requires a high degree of clinical suspicion and diagnostic acumen (34).

Prompt airway management, including orotracheal or surgical intervention, is paramount in laryngotracheal injuries (29). Although prompt diagnosis and surgical repair are considered the classical approach, nonoperative management has gained traction for specific tracheal injuries, showcasing successful outcomes in carefully selected patients (35). While surgical intervention remains the standard for larger tracheal lacerations or those with clinical instability signs, some extensive tracheal lacerations have been managed successfully with antibiotics and observation alone (35). The management of major tracheobronchial injuries often necessitates a high level of suspicion and surgical expertise tailored to the specific injury (36). Early and organized diagnosis coupled with classification is critical for early management, optimizing function and reducing morbidity.

### Tracheostomy decannulation

#### Assessment for decannulation readiness

Decannulation, or the removal of the tracheostomy tube, is a significant milestone for children with tracheostomies. The decision to decannulate should be based on a comprehensive assessment of the child's respiratory status, airway patency, and overall medical condition. Factors to consider include the resolution of the underlying medical condition that necessitated the tracheostomy, the child's ability to protect their airway, the presence of any airway obstruction or instability, and the child's tolerance of upper airway airflow. Pulmonary function tests, flexible bronchoscopy, and overnight oximetry studies may be performed to assess the child's respiratory function and airway patency (37). A crucial part of the decannulation process involves evaluation by a multidisciplinary team, including otolaryngologists, pulmonologists, and speech therapists.

#### Decannulation techniques

Decannulation techniques vary depending on the child's age, medical condition, and the duration of tracheostomy. The process involves downsizing the tracheostomy tube over a period of time, until the tube is plugged for a period of 24 h. If the patient tolerates the capping period without any respiratory distress or other problems, the tracheostomy tube can then be removed entirely. After decannulation, the stoma usually closes on its own within a few days. Some surgeons prefer to close the stoma surgically, but the evidence does not suggest a difference between the two options.

#### Post-decannulation care

After decannulation, close monitoring of the child's respiratory status is essential to ensure adequate oxygenation and ventilation. Parents and caregivers should receive comprehensive education on stoma care, signs of respiratory distress, and emergency procedures. Some studies suggest that adhering to a standardized protocol helps reduce extubation failure and increases patient recovery (38). Home nursing needs may be inadequately met in some families, leading to difficulties soon after discharge, and a significant percentage of families may seek emergent care within a week of discharge (39). The success rate in decannulation or the resolution of respiratory failure with discharge from the ICU, after the procedures for correcting stenosis in childhood, can be as high as 94.4% (40).

### Long-term outcomes and quality of life

#### Speech and language development

Tracheostomy can impact speech and language development in children, particularly if performed at a young age (41). The presence of a tracheostomy tube alters the normal airflow patterns required for speech production, which can lead to delayed speech development, articulation difficulties, and voice abnormalities. Speech therapy plays a crucial role in helping children with tracheostomies develop their communication skills. Early intervention and specialized techniques can help children overcome these challenges and achieve their full communication potential.

#### Swallowing and feeding difficulties

Children with tracheostomies are at risk for swallowing and feeding difficulties, particularly those with underlying neurological or anatomical abnormalities (42). The presence of a tracheostomy tube can interfere with the normal swallowing mechanism, leading to aspiration, dysphagia, and feeding aversion. A multidisciplinary approach, involving speech therapists, occupational therapists, and dietitians, is essential for managing these challenges and ensuring adequate nutrition.

#### Psychological and social impact

The presence of a tracheostomy can have a significant psychological and social impact on children and their families. Children with tracheostomies may experience anxiety, depression, and social isolation due to their altered appearance and communication difficulties. Parents and caregivers may also experience stress, burden, and emotional distress related to the care of their child. Support groups, counseling, and peer support can help children and families cope with these challenges and improve their overall quality of life.

#### Swallowing function

About 80% of children with tracheostomies experience dysphagia (42). Some studies suggest that early and aggressive management of swallowing dysfunction, including trials of oral feeding and bolus modification, is safe and effective.

## Summary of key findings

Pediatric tracheostomy is a complex procedure with evolving indications, techniques, and management strategies. While it can be a life-saving intervention for children with airway obstruction or respiratory failure, it is associated with significant risks and long-term consequences. Advancements in surgical techniques, postoperative care, and multidisciplinary management have improved outcomes and quality of life for children with tracheostomies. Ongoing research is needed to further refine best practices, minimize complications, and optimize long-term outcomes for this vulnerable population.

Esophageal atresia and tracheoesophageal fistula are congenital malformations frequently encountered, and the molecular mechanisms remain under investigation (43). Acquired tracheo-esophageal fistula is very infrequent in the pediatric age group and is usually caused by esophageal foreign body impaction (44). Isolated tracheo-esophageal fistula presents with choking and cyanosis on feeding, recurrent lower respiratory tract infection and abdominal distension (45).

## Implications for clinical practice

The management of pediatric tracheostomy requires a multidisciplinary approach, involving surgeons, pulmonologists, nurses, speech therapists, and other healthcare professionals. Careful patient selection, meticulous surgical technique, and comprehensive postoperative care are essential for minimizing complications and optimizing outcomes. Protocols for tracheostomy care, decannulation, and long-term follow-up should be established and regularly updated based on the best available evidence. Timely and accurate diagnosis of conditions necessitating tracheostomy, followed by prompt surgical intervention when appropriate, are crucial for preventing further morbidity and optimizing patient outcomes (46).
